# Transport Infrastructure Surveillance and Monitoring by Electromagnetic Sensing: The ISTIMES Project

**DOI:** 10.3390/s101210620

**Published:** 2010-11-29

**Authors:** Monica Proto, Massimo Bavusi, Romeo Bernini, Lorenzo Bigagli, Marie Bost, Frédrèric. Bourquin, Louis-Marie Cottineau, Vincenzo Cuomo, Pietro Della Vecchia, Mauro Dolce, Jean Dumoulin, Lev Eppelbaum, Gianfranco Fornaro, Mats Gustafsson, Johannes Hugenschmidt, Peter Kaspersen, Hyunwook Kim, Vincenzo Lapenna, Mario Leggio, Antonio Loperte, Paolo Mazzetti, Claudio Moroni, Stefano Nativi, Sven Nordebo, Fabrizio Pacini, Angelo Palombo, Simone Pascucci, Angela Perrone, Stefano Pignatti, Felice Carlo Ponzo, Enzo Rizzo, Francesco Soldovieri, Fédrèric Taillade

**Affiliations:** 1 Consiglio Nazionale delle Ricerche, Istituto di Metodologie per l’Analisi Ambientale (IMAA), Tito Scalo (PZ), Italy; E-Mails: proto@imaa.cnr.it (M.P.); bavusi@imaa.cnr.it (M.B.); bigagli@imaa.cnr.it (L.B); cuomo@imaa.cnr.it (V.C.); lapenna@imaa.cnr.it (V.L.); loperte@imaa.cnr.it (A.L.); mazzetti@imaa.cnr.it (P.M.); nativi@imaa.cnr.it (S.N.); palombo@imaa.cnr.it (A.P.); pascucci@imaa.cnr.it (S.P.); perrone@imaa.cnr.it (A.P.); pignatti@imaa.cnr.it (S.P.); rizzo@imaa.cnr.it (E.R.); 2 Tecnologie per l’Osservazione della Terra e dei Rischi Naturali (TeRN) Consortium, Tito Scalo (PZ), Italy; 3 Consiglio Nazionale delle Ricerche, Istituto per il Rilevamento Elettromagnetico dell’Ambiente (IREA), Naples, Italy; E-Mails: bernini.r@irea.cnr.it (R.B.); fornaro.g@irea.cnr.it (G.F.); 4 Laboratoire Central des Ponts et Chaussées (LCPC), Paris, France ; E-Mails: bost@lcpc.fr (M.B.); bourquin@lcpc.fr (F.B.); louis-marie.cottineau@lcpc.fr (L.M.C.); dumoulin@lcpc.fr (J.D.); taillade@lcpc.fr (F.T.); 5 Territorial Data Elaboration (TDE), Bucaresti, Romania, E-Mail: comercial@tde.ro (P.D.V.); 6 Dipartimento della Protezione Civile (DPC), Roma, Italy; E-Mails: segreteriacapodipartimento@protezionecivile.it (M.D.); Claudio.Moroni@protezionecivile.it (C.M.); 7 Department of Geophysics and Planetary Sciences, Tel Aviv University (TAU), Tel Aviv, Israel; E-Mail: levap@post.tau.ac.il (L.E.); 8 Lund University (ULUND), Lund, Sweden; E-Mail: Mats.Gustafsson@eit.lth.se (M.G); 9 Eidgenoessische Materialpruefungs-und Forschungsanstalt (EMPA), Duebendorf, Switzerland; E-Mails: Johannes.hugenschmidt@empa.ch (J.H.); Hyunwook.Kim@empa.ch (H.K.); 10 Norsk Elektro Optikk (NEO), Lorenskog, Norway; E-Mail: peter@neo.no (P.K.); 11 Elsag Datamat (ED), Genova (GE), Italy; E-Mails: Fabrizio.Pacini@elsagdatamat.com (F.P.); Mario.Leggio@elsagdatamat.com (M.L.); 12 Linnaeus University, Växjö, Sweden; E-Mail: sven.nordebo@lnu.se (S.N.); 13 Università degli Studi della Basilicata, Dipartimento di Strutture, Geotecnica, Geologia Applicata all’Ingegneria (DiSGG)-Potenza, Italy; E-Mail: felice.ponzo@unibas.it (F.C.P.)

**Keywords:** electromagnetic sensing techniques, system architecture, transport infrastructure non invasive monitoring

## Abstract

The ISTIMES project, funded by the European Commission in the frame of a joint Call “ICT and Security” of the Seventh Framework Programme, is presented and preliminary research results are discussed. The main objective of the ISTIMES project is to design, assess and promote an Information and Communication Technologies (ICT)-based system, exploiting distributed and local sensors, for non-destructive electromagnetic monitoring of critical transport infrastructures. The integration of electromagnetic technologies with new ICT information and telecommunications systems enables remotely controlled monitoring and surveillance and real time data imaging of the critical transport infrastructures. The project exploits different non-invasive imaging technologies based on electromagnetic sensing (optic fiber sensors, Synthetic Aperture Radar satellite platform based, hyperspectral spectroscopy, Infrared thermography, Ground Penetrating Radar-, low-frequency geophysical techniques, Ground based systems for displacement monitoring). In this paper, we show the preliminary results arising from the GPR and infrared thermographic measurements carried out on the Musmeci bridge in Potenza, located in a highly seismic area of the Apennine chain (Southern Italy) and representing one of the test beds of the project.

## 1. Introduction

Terrorist actions and natural disasters often endanger civil and critical infrastructures of strategic interest, making them strongly vulnerable. As well known, the surface transportation system consists of interconnected infrastructures including highways, transit systems, railroads, airports, waterways, pipelines and ports, and it involves different types of vehicles, aircraft, and vessels that operate along these networks [1,2].

In this framework, the research project for “Integrated System for Transport Infrastructure surveillance and Monitoring by Electromagnetic Sensing” (ISTIMES) has been submitted in the 7th Framework Programme and approved by the European Commission. It is started on 1st July 2009 and has a three year duration.

The main aim of the project is to design, assess and promote an Information and Communication Technologies (ICT)—based system, exploiting distributed and local sensors, for non-destructive electromagnetic monitoring in order to achieve safer and more reliable critical transport infrastructures. This takes the overall aim at developing a high situation awareness in order to provide real time and detailed information and images of the infrastructure status to improve decision support for emergency and disasters responders.

**Figure 1.**
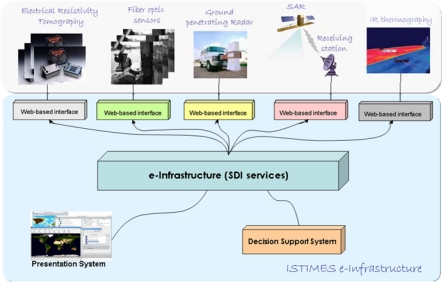
The ISTIMES system architecture: The integration of electromagnetic technologies with new ICT information and telecommunications systems.

The system exploits an open network architecture (see Figure 1) that can accommodate a wide range of sensors, both static and mobile, and can be easily scaled up to allow the integration of additional sensors and interfacing with other networks. It relies on heterogeneous state-of-the-art electromagnetic sensors, enabling a self-organizing, self-healing, *ad-hoc* networking of *terrestrial* sensors, supported by specific *satellite* measurements. The integration of electromagnetic technologies with new ICT information and telecommunications systems enables remotely controlled monitoring and surveillance and real time data imaging of the critical transport infrastructures.

Thus, the proposal also concerns with the development of tools for handling, analysing and processing large data volume (Information Fusion) and then providing information and performing behaviour prediction in a quick, easy and intuitive way (Situation Awareness).

The project is carried out by a consortium composed by nine partners, all having a long experience in security issues, electromagnetic sensing of the environment and of civil infrastructure, as well as in the development of expert systems for data harmonization and data access service interoperability. They consist of universities, Lund University (SW) and Tel Aviv University (IL), technology research institutions, Eidgenoessische Materialpruefungs und Forschungsanstalt (CH) and the French Public Works Research Laboratory (FR), the Italian Civil Protection Department (IT) and some high technologies companies specialized in risk management, sensor engineering and information communication technologies such as Elsag Datamat (IT), T.D.E.-Territorial Data Elaboration (RO), Norsk Elektro Optikk A/S (NO). The coordinator of the project is the Earth Observation and Natural Hazard Technologies Consortium, whose main activities are focused on the development of innovative technologies and methodologies aimed to the protection and prevention of environmental and natural hazards.

Some preliminary results of the project are already available. Here, we show the exploitation of electromagnetic sensing techniques as Ground Penetrating Radar (GPR) and Infrared Thermography (ThIR) at one of the bed-sites selected in the project, the Musmeci bridge, located in a highly seismic area, the Basilicata Region (Southern Italy). In this paper, as scientific outcome of the work, we will present the reconstruction results form GPR data allowing detection of the rebars embedded both in the shallower and in the deeper layers of the concrete of the bridge. Finally, images of the overall structure based on the ThIR will be shown.

## 2. Methodology and Techniques

In order to monitor critical transport infrastructures, *in situ* sensors networks as well as their integration with remotely sensed data represent a key point from both economic and safety viewpoint. To date, data from *in situ* monitoring sensors and remote sensing systems are mostly acquired and managed independently by different organizations and are characterized by different data models and access services. Hence, the proposed integrated system implements and experiments solutions for data harmonization and data access service interoperability in the context of Civil Infrastructures Monitoring by Electromagnetic Sensing techniques.

### 2.1. Electromagnetic Sensing Techniques

The proposal aims at developing an integrated monitoring and surveillance system able to collect, treat and combine the information acquired by *in situ* and remote sensors based on electromagnetic sensing. Sensing techniques operating at different spatial and time scales are thus used in conjunction to improve the capabilities and performances of the monitoring system for security/safety/management issues.

**Figure 2.**
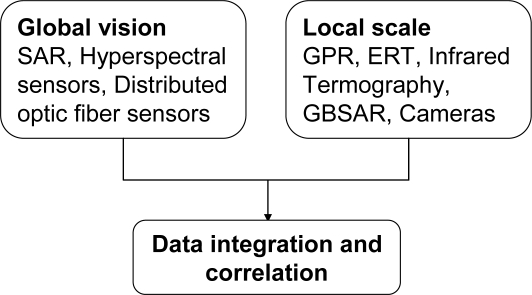
Electromagnetic sensing techniques classified in the two main classes of global vision and local scale techniques.

Core sensing systems concerning structure monitoring are Synthetic Aperture Radar satellite based platform, Hyperspectral spectroscopy, Distributed Optic Fiber sensors, Electrical Resistivity Tomography, Ground Penetrating Radar systems, Infrared Thermography, Ground based systems for displacement monitoring. These ones can be grouped in “global” vision techniques and “local” scale techniques (see Figure 2). The former are able to monitor wide areas including ground deformation, the overall infrastructure (its status included) and the surrounding environment, while the latter can provide insight on the status of the infrastructure itself during the normal operations and after the crisis event.

Satellite remote sensing with *Synthetic Aperture Radar (SAR)* is a key technique for the monitoring of ground deformation associated to natural risks and for the stability of structures and infrastructures. Multi-temporal observations will be performed and the data via multi-pass Differential Interferometric SAR (DInSAR) techniques will be processed, allowing the processing of data stacks corresponding to the different acquisitions. The available DInSAR software has been derived in house and allows an end-to-end processing from the raw data up to the target time series. The algorithm has been already validated via comparison with traditional leveling and GPS and widely tested in many areas of natural risk management as volcanoes, earthquakes, underground extractions (water, oil, *etc*.), slope instabilities.

In the ISTIMES project, the monitoring of transport infrastructures will be carried out at highest possible spatial resolution via the use of advanced multidimensional imaging techniques [3–7] which allow extending traditional methods based on Persistent Scatterers Interferometry. It allows carrying out accurate tracking of the slow movement of ground targets and even separating the information from targets interfering in the same image pixel. Such a technique has been recently patented [8]. *Hyperspectral spectroscopy* in the Visible, Near InfraRed (VNIR) and ShortWave InfraRed (SWIR) spectral ranges, is a fast and non-destructive method, which allows one to provide potentially useful alternatives to time-consuming methods of surfacing materials analysis [9]. The innovative aspect of this activity is the development, implementation and validation of the hyperspectral spectroscopy, in the VNIR/SWIR spectral range, to characterize the condition of the pavement (road/railway) and of the surrounding environment that can be related to the infrastructure status [10–13]. In order to evaluate the pavement conditions a spectral library of diverse typology of surface (asphalt/concrete/soil) under different conditions (*i.e.*, factors that can modify the surface reflectance) will be built using a FieldSpec Vis/NIR full-range portable spectrometer; remote hyperspectral surveys will be planned and carried out on the base of the SAR infrastructure status analysis results (e.g., indication of structural differential shifts) and spectral indexes will be applied for monitoring purposes.

*Distributed Optic Fiber Sensors (DOF)* based on Brillouin scattering phenomenon is an *in situ* sensing technique. Unlike other fiber optic sensors, this technique permits the remote and *spatially continuous* monitoring of the structure in terms of temperature and strain with the resolution of some metres [14,15]. Usually, distributed fiber optics sensors based on Brillouin scattering works in time-domain (BOTDA). For such schemes, some difficulties in the acquisition could occur, such as the limited spatial resolution, loss of accuracy of the sensor in specific situations, and the negative effects due to the so-called non-locality effects. The innovative instrumentation, measurement methodologies and reconstruction algorithms that process the data in frequency domain have been developed [15,16] and will be applied to the case study of the project, improving the results and allowing to overcome some of the difficulties of the classical BOTDA techniques.

The *Electrical Resistivity Tomography (ERT)* method is widely applied to obtain high resolution images of the subsurface in areas of complex geology [17–24]. Significant advances have been made with PC-controlled data acquisition systems, using multi-channel and multi-sensor measurements. Recently, the combination of new technologies for data acquisition, based on multi-channel systems and microsensors, discloses the way for applying the ERT in more specific engineering problems. In particular, great attention has been devoted to the application of micro ERT to diagnose civil infrastructures, such as pillars, buildings, dam embankment, *etc.* The micro ERT can be applied to detect and locate, at small spatial scale, possible anomalies in the structure order. Moreover, time-continuous 2D and/or 3D micro ERTs allow to monitor in near-real time the state of the structural anomalies and to define the thresholds for early warning. However, the need to insert steel electrodes for galvanic contact normally precludes the use of this technique on engineered structures or surfaces (e.g.,: concrete, masonry and other artificial materials). Such materials cannot be invasively examined, hence we propose to exploit a capacitive resistivity system for permanent *in situ* ERT monitoring. An adhesive will be used to attach these non-contacting plate electrodes to the engineered surface. Volumetric images of the internal resistivity distribution will be obtained by using existing 2D, 3D and time-lapse 4D ERT inversion schemes. Also, new and more flexible Finite Element algorithms will be needed to cope with the irregular geometry of engineered structures. New research will also be undertaken to relate the derived geoelectric property variations to geotechnical properties or processes using established mathematical and empirical relationships.

The *Ground Penetrating Radar (GPR)* technique works by generating and radiating several short pulses (pulsed GPR) or, alternatively, several time harmonic waves (stepped frequency GPR) towards a target medium [25,26]. When the transmitted wave hits an object with different electromagnetic properties (dielectric permittivity, conductivity) a backscattered electromagnetic wave arises and is recorded at a receiving antenna. Since the backscattered wave contains information about the electromagnetic properties of the object, suitable processing can lead to reveal the presence and the location of the target as well as to characterize its properties. A growing interest concerns the analysis and the experimental validation of innovative reconstruction algorithm based on inverse scattering problem [27,28], able to provide a spatial map of the dielectric permittivity and/or of the electrical conductivity of a subsurface region that gives a detailed image of the inner status of the structure under test, with a resolution in the centimetre range [29,30]. This permits to achieve information not only about the presence and the position of the object but also about its extent and shape. The other field of interest is the development of a novel system for automated data acquisition based on a vehicle that is travelling all the time or frequently on roads for other reasons, such as a regular bus or a lorry. This will enable a 4-D monitoring of structures and thus bridge the gap between non-destructive testing and monitoring.

In the frame of the ISTIMES project, the activities will be mainly addressed to: (a) the development of a novel system for automated data acquisition based on a vehicle that is travelling all the time or frequently on roads for other reasons, such as a regular bus or a lorry; (b) the exploitation of prototypical measurement instrumentation and configurations and innovative processing algorithms based on accurate models of the electromagnetic scattering combined with linear inversion regularized schemes (Singular Value Decomposition) [27,29] and tools and methods from statistical signal processing (Fisher information and Bayesian estimation) [28,30]. The overall aim is to achieve images of the inner status of the structure clearer, more objective and at a better resolution with respect to the ones achieved by adopting the usual radar techniques.

Another *in situ* sensing technique for the high resolution diagnostics of the shallower part of the infrastructure as concrete and asphalt roadbeds is *Infrared thermography (ThIR).* ThIR performance relies on the knowledge of the thermo-optical properties of the observed scene [31], the extinction of the signal in the atmosphere and the infrared camera (detector and optics) characteristics [32]. Of course, suitable inverse modelling is needed to reject perturbations due to environmental variations and to get information on the thermal state inside the structure. Quasi real-time inversion [33] is an issue since it can be proved that observers of Kalman type cannot work to that purpose. But least-squares formulated in spaces of sufficiently smooth functions enables to reconstruct current temperature field by mean of past measurements at several points.

In the ISTIMES project, the main objectives of the activity for this technique are:

1. the qualification of low cost uncooled infrared array camera performances and evaluation of the influence of emissivity properties (to be measured), atmosphere transmission (to be determined) and location from the scene (to be simulated) on infrastructure surface temperature measurement in survey configuration.
2. the validation of different models and solutions to correct surface temperature measurement using an infrared uncooled array camera used for infrastructure survey in various weather conditions and at different distance from the scene.
3. the development and exploitation of innovative processing algorithms with particular attention to a situation where a large number of data is available only on part of the boundary of the structure;
4. the improvement of the inversion algorithms to permit the processing of very large amount of data thanks to the exploitation of intelligent reduction methods and optimized algorithms.

*Ground-based SAR (GBSAR)* is an active sensor working in the microwave range. It is made up of a ground based interferometric sensor operating on the same principles of satellite platform based ones. The high resolution capacity over distance provided by the radar produces a displacement map. Thanks to the microwave technology used, the radar can detect displacements of each point on the target up to an oscillation frequency of 50 Hz with a detection limit of less than 1/10 mm. During the project, a novel instrumentation [34,35], of recent acquisition by TERN will be exploited for monitoring the infrastructures of interest, with a particular attention to the structures involved in the demonstration activities.

*Ground based stationary image based displacement monitoring* will be developed and exploited during the project. It is an image based displacement monitoring system based on a high resolution camera depicting an area where distinct point light sources are mounted on reference points. The camera with weather protection facilities is mounted on a firm foundation while the point light sources will be mounted on reference points within the camera’s field of view.

### 2.2. System Architecture

**Figure 3.**
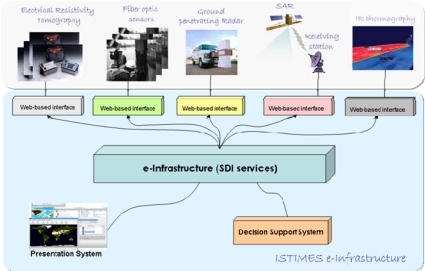
ISTIMES architecture based on web sensors and service-oriented-technologies.

The architecture will be based on web sensors and service-oriented-technologies that comply with specific end-user requirements, including economical convenience, exportability, efficiency and reliability. The system will adopt open architectures and will make efforts to achieve full interoperability, taking into account the relevant international initiatives and programmes for the standardization of geospatial information, such as the INSPIRE Directive (http://inspire.jrc.ec.europa.eu/index.cfm), the ISO Technical Committee 211 (http://www.isotc211.org/) and the Open Geospatial Consortium (http://www.opengeospatial.org/). The ISTIMES infrastructure shown in Figure 3 is made up of the following main components:

- *Web-based interface components* for the corresponding kinds of the electromagnetic sensors exploited in the project;
- *an e-Infrastructure* for geospatial data sharing;
- *a Decision Support component* which helps decision makers providing inferences and situation indexes;
- *a Presentation component* which implements system-users interaction services and information publication and rendering.

The *Web-based interface* for sensors and sensor constellation allows the sensors to be accessible and controllable via the World Wide Web. These components, one for each type of sensor or sensor constellation, implement services in order to:

- control sensor or sensors constellation, defining acquisition policies; discover sensors and get metadata about sensor/sensor constellation data model and services interface;
- access sensor or sensor constellation measurements and observations, using either synchronous or asynchronous solutions;
- implement security functionalities, such as: authentication, authorization, audit, *etc.*

These services are accessed through standard Web interface, using either service-oriented or resource-oriented approaches.

The *e-Infrastructure* implements the main services of a SDI (Spatial Data Infrastructure) for geospatial data sharing:

- distributed and transparent discovery;
- access;
- browsing and evaluation.

The *Decision Support System* implements the following services and functionalities:

- data fusion;
- inference engine, on the top of a knowledge model;
- situation indexes generation.

The *Presentation System* implements the following services:

- user interface to allow interactions;
- publication of situations.

## 3. Research Activities

**Figure 4.**
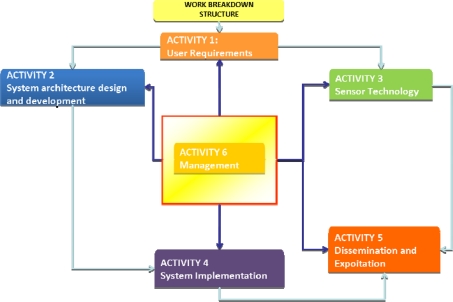
Organization of the ISTIMES project in six activities.

The ISTIMES project is organized in six activities, four of these mainly are concerned with research and technological development and the other with management and dissemination (see Figure 4).

As the ISTIMES project concerns with the security and the safety of strategic infrastructures, the first activities are addressed to the definition of the user requirements dealing with both domain and system, in order to describe the application context and the user and system interactions. It is important, in this phase, to collect the required expertise both in the scientific domain and in the ICT applications, in order to achieve a detailed description of the user-system interactions and a view of the existing technological resources and infrastructures.

On the basis of the kind of information provided by the previous activity and the state of art about the main European and international initiatives concerning with the interoperability and standardization of system architectures, the ISTIMES e-infrastructure will be designed, taking into account different needs, such as those of ensuring that the system is coherent, satisfies the needs of the user community and properly utilizes current standards. The ISTIMES infrastructure has to guarantee a real time and interactive access to the information by end-user, a remote use and control of instrumentation and processing of measurements and has to include wireless network services for sensors communication.

Besides that, the activities aim at defining the system architecture of the ISTIMES environment, to provide contribution to existing standardization initiatives in the field of web services for sensor management and access. The other objective is to provide a general framework (common functionalities and common interfaces) to implement monitoring services based on sensor networks.

This kind of architecture is a complex system which must be carefully designed and then developed and tested. It is necessary to implement each single system component, test it and then integrate each other. The integration and implementation of monitoring results will be managed through a Geographic Information System (GIS) database. The final result will be the deployment and test of the system.

As regards to the sensor technologies, it is important to improve, exploit and integrate innovative processing approaches and measurement strategies for non invasive monitoring of the structures in order to ensure a multi-temporal and multi-spatial scale monitoring with different levels of resolution. This overall objective is pursued in particular with the development and the integration of different electromagnetic sensing techniques all based on non- or minimally invasive diagnosis. Besides the exploitation and development of each electromagnetic sensing technique, the challenge of the ISTIMES project is to integrate them and finally to validate them in controlled conditions thanks to state-of art and innovative test sites available at the partners’. In particular, during the ISTIMES project experiments in controlled conditions will be performed in order to analyze the skills of each EM technologies.

**Figure 5.**
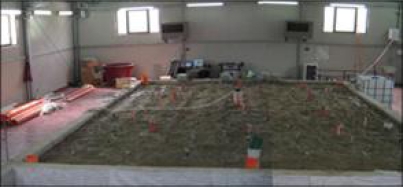
HYDROGEOSITE: A new laboratory at TERN (CNR-IMAA) in Marsico Nuovo (PZ, Southern Italy).

Two test sites are available to the ISTIMES partners, one in Italy at TERN and the other in France at LCPC. The first test-site at TERN (see Figure 5) represents an intermediate stage between laboratory experiments and a field survey. Therefore, it has the advantage of providing controlled results, like in a laboratory experiment, but at scales comparable to the field ones. It consists of a pool (12 × 7 × 3 m) completely covered with a steel shed. Using a regular grid of 64 close holes, it is possible to take samples and/or to install sensors without disturbing the surface of soil. The pool is filled with fine quartz sand with a filter on the bottom to permit a controlled discharge of water. The sand is a homogeneous siliceous sand (95% of SiO_2_), which has a 87% percent of granulometry between 0.063 mm to 0.125 mm and a permeability of about 4.10–3 cm/s. *In situ* techniques such as ERT, Infrared Thermography GPR and Fiber Optics sensors (already installed in the pool) will be validated in a test case simulating road and subsurface diagnosis. At this test site, we will perform experiments simulating the *in situ* monitoring of a bridge deck made up of different layers, rebar and utilities (metallic and non-metallic pipes). The experiments will be designed to perform the validation of the *in situ* sensing techniques such as: Hyperspectral spectroscopy; Fiber Optic Sensors; Electrical Resistivity Tomography, Ground Penetrating Radar; Infrared Thermography.

**Figure 6.**
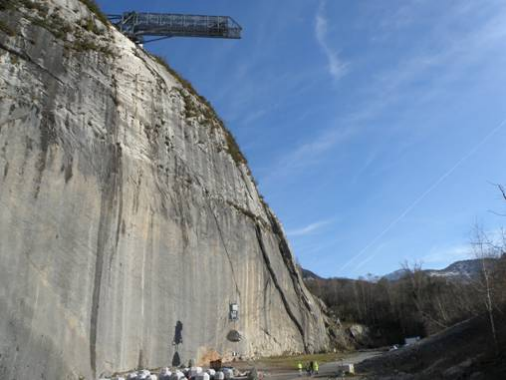
Global view of the LCPC rockfall test station.

The second test-site is the French falling block station (Montagnole, French Alpes) owned by LCPC. This new testing station located in French Alps is able to drop heavy loads (up to 20 tons) from the top of a cliff down to structural systems in order to test their resistance to big shocks and study their dynamic behavior at this high energy level (Figure 6). As the fall height can reach near 70 m, the impact velocity can actually reach 35 metres per second and the energy released during the impact can be as large as 13,500 kilojoules. The experimental area at the bottom of the cliff which can be impacted by a block is half disk of 12 metres radius. This configuration allows testing of different parts of building structures against impact and special protection systems at scale 1. This allows one to test large scale structures that may even be slanted. The safety of the staff is ensured by strict operating rules and a big external earth wall. Observation platforms set uphills will enable monitoring the experiments.

For the ISTIMES project, the station will be used to compare the skills of the different studied EM technologies. Some experiments will be performed on an instrumented structure under controlled conditions. First a long cement concrete beam laid on two supports has been chosen to be tested like a structure. The controlled solicitations will be at the beginning indirect impacts on the ground not too far from the structure. Direct impacts on the structure will be then performed at increasing impact energy level in order to load progressively the structure to its failure. These different tests will allow to experimentally validating the different sensing techniques in controlled condition and evaluating the synergic effect of the jointly application of all the EM technologies.

Other interesting activities that will be performed during the ISTIMES project concern with the implementation of the system infrastructure, that will be applied on specific critical structures, in order to demonstrate the effectiveness of the integrated and distributed monitoring system. For this purpose, two test beds with significant end-users requirements have been selected, the Sihlhochstrasse highway-bridge in Zurich (Switzerland) and railway and highway infrastructures in the Basilicata region (Italy). The demonstration of the effectiveness of the overall monitoring system at these two different engineered sites, pose very challenging issues, because the first one has a structural complexity and the other is located in a high seismic risk affected area

**Figure 7.**
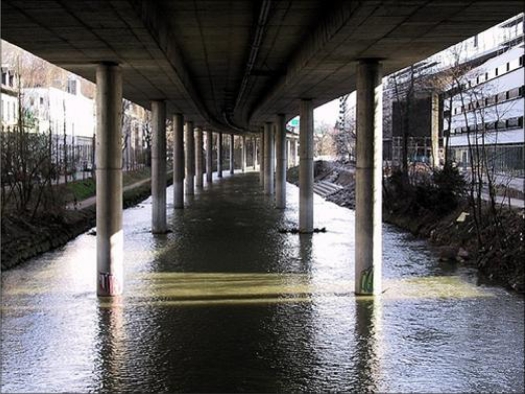
The Sihlhochstrasse bridge selected as bed site for its size and its structural complexity.

The Sihlhochstrasse bridge was built in 1973 and is located in Zurich (see Figure 7). It is one of the largest bridges in Switzerland with a length of 1.5 km. Its size and its structural complexity represent a challenging test bed for the ISTIMES system. Empa is currently carrying out several bridge-related research projects in cooperation with Swiss Federal Roads Authority (Fedro). Many of those projects include work on existing bridges. Fedro has taken over the responsibility for all motorway bridges in Switzerland on January 1, 2008. The collaboration between EMPA and FEDRO allowed to use Sihlhochstrasse bridge for testing and demonstration purposes.

**Figure 8.**
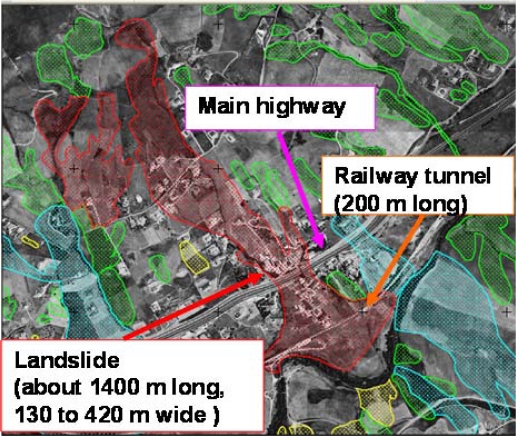
Varco d’Izzo test bed, an area located near Potenza city (Southern ITALY) and affected by a high hydro-geologic risk.

The other bed site is the railway tunnel located along the Potenza—Metaponto line in Basilicata Region (Southern Italy), the only one and important section of the Napoli-Potenza-Taranto railway (see Figure 8). The line skirts the Basento River for several kilometres as far as Metaponto town, on the Ionian Sea. Along its route, two critical situations were identified. Firstly, the railway is involved for about 500 m in a landslide occurring in Varco d’Izzo site in the territory of Potenza City [36]. The mass movement, classified as an active rototranslational-slide, is one of the most dangerous mass movements of the whole Lucanian Apennine Chain. In particular, the stretch of railway involved in the landslide is constituted, for a length of 200 m by a railway tunnel, which is completely included in the unstable area.

Moreover, the landslide involves a stretch of a main highway: the SS 407 Basentana and several residences and secondary roads. Basentana highway, involved in the movement for about 130 m, is a strategic life line for Basilicata Region because it links two capitals of province, Potenza and Matera cities, and the main regional industrial areas of Ferrandina and Pisticci towns.

**Figure 9.**
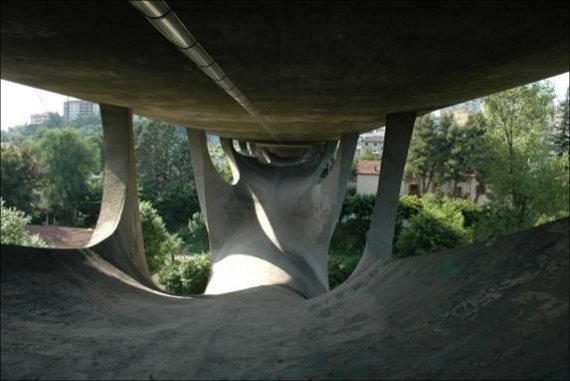
Musmeci Bridge in Potenza, (Southern ITALY), a highly seismic area.

A few kilometres from the above railway tunnel, an other critical point has been individuated: it is the Musmeci bridge (Figure 9). It represents an important flyover 300 m long linking the city to the link road Potenza-Sicignano. This bridge planned by Sergio Musmeci (1926–1981) in the ’60, is a true work of art which influenced the architectonic culture of the XXth century.

At present, the bridge, constituted by reinforced concrete, shows related aging problems and causes worries related to the recent growth of the traffic load. A monitoring plan of this important structure having a direct impact on the lower railway line is necessary.

The above depicted situations assume greater importance if we consider that they are located in Basilicata Region, one of the most seismically active and subject to hydro-geological risk of all Italian regions.

As stated in the ISTIMES project, two levels of observations should be applied, one based on *in situ* techniques, the other on the use of satellite and airborne observations in order to monitor the surface of the infrastructures and the surrounding areas. The outcomes of these remote observations will be supported by the data obtained by using ground-based techniques. In this way, a more detailed study of the infrastructures and of more important close areas will be performed. In particular, for both the structures, the activities will be concerned with the deformation monitoring, the surface analysis and the check of the inner of the structures in order to locate possible weak points. This will be performed thanks to the exploitation of the some of the *in situ* techniques such as DOF, GPR, ERT, CRI and THiR.

## 4. Preliminary Results

**Figure 10.**
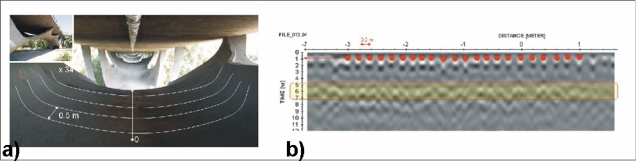
**(a)** The Musumeci Bridge (Potenza City, Italy) and its lower structure with the traces of a GPR survey; **(b)** a radargram showing the position of shallow rebars (red dot) and deep rebars (rectangle).

A first survey of measurements on Musmeci bridge was carried out on February 2010, using two different *in situ* techniques such as GPR and ThIR. As regards the GPR, a GSSI SIR3000 unit equipped with a 400 MHz central frequency antenna was used, with a time range limited to 40 ns. Moreover, low and high pass filters were set at 800 MHz and 66 MHz, respectively. Field gains were set up on four points. In order to detect rebars embedded in a longitudinal way to the bridge axis, the radargrams were gathered by sectioning the bridge transversally [Figure 10(a)].

**Figure 11.**
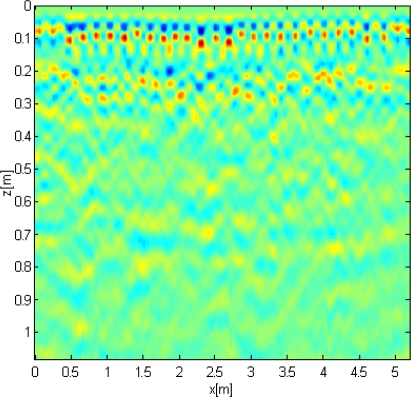
Raw GPR data acquired on Musmeci Bridge.

Data processing steps were: trace editing, marker interpolation, time zero correction, header gain removal, ACG gain and fk-filter. Finally, data were migrated with a velocity of 0.06 m/ns estimated by using the hyperbola adaptation method. Although the survey was performed in a dry period, the encountered value of velocity can be indicative of a great moisture content of the concrete. Figure 11 shows an example of raw data obtained on Musmeci bridge, with the time-zero stated at 3.5 ns in order to eliminate the unperturbed field due to the air/soil boundary. It is possible to detect single rebars of the shallower layer but the second and deeper ones appear blurred and it is not possible to clearly distinguish them.

**Figure 12.**
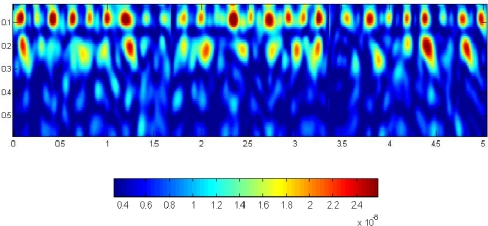
Microwave tomographic reconstruction of he raw GPR data shown in Figure 11.

In order to focus the second layer, a novel inversion algorithm was applied: the *Microwave Tomography*; in particular, the approach followed in this work is based on the Born Approximation (BA) [29,37,38]. The unknown of the problem is the *contrast function* obtained by the scattered field in the frequency domain. The scattered field is obtained by subtracting the unperturbed field by the total field in the time domain. In the case of the homogeneous semi-space, unperturbed field is just the air/soil boundary. The spatial reconstruction of the modulus of the contrast function can be considered equivalent to a focalization of a radargram [27,29]. Figure 12 shows the result obtained by applying the MT to the raw data of Figure 11. It shows the anomalies in terms of contrast function. Maximum zones bring information about the location of rebars. The algorithm exploits all available spatial resolution providing a precise focalization of the deeper rebar layer. Moreover, other advantages related to the MT processing approach consist of the possibility to obtain more objective results than the classical processing approach in a fast and automatic approach.

At the same time of GPR surveys, measurements based on the Infrared Thermography (ThIR) were performed on Musmeci bridge. A relatively low cost thermal camera (FLIR SC-7000) covering the 8–12 μm spectral range has been used in order to obtain very high spatial resolution thermal imagery, which is advantageous for four reasons: (1) there is a reduction of uncertainty introduced by subpixel mixing; (2) limitation to a local scale improves the assumption that meteorological variables can be measured on site and have a high degree of spatial uniformity; (3) differences between single- or dual-source and mosaic patch models become smaller as pixel size approaches the scale of heterogeneity; and (4) detailed variation in thermal patterns that would be lost with coarser resolution can be observed and can help provide an understanding of the process, distribution, and nature of asphalt surface defects.

The objectives of this research are to develop a cost effective and transferable methodology for mapping site-scale, daily temperature variations at a very high spatial resolution and to apply it in a comparative analysis with the other technologies used and proposed in this study.

The main characteristics of the camera are: FPA: MCT; Sensor pitch: 320 × 240; spectral response: 8–12 μm; full frame rate: Up to 380 Hz; integration time: 1 μs to 20 ms variable by 1μs step; NETD: <20 mK; Filter wheel: Motorized, 4 slots for 1” filters, up to 2.5 mm thick.

**Figure 13.**
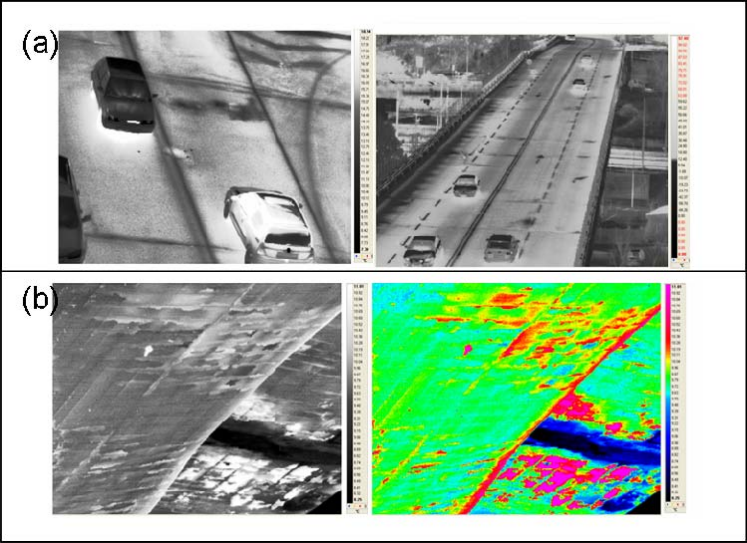
Temperature images acquired on Musmeci Bridge by FLIR SC-7000 thermo cam. **(a)** Shows the entire thermal imagery acquired on the bridge at a height of 40 m; **(b)** examples of images acquired under the bridge.

The FLIR camera was mounted on a crane at 40 m of height on the bridge and was maintained in a near vertical orientation (<10- deviation) with a manually controlled tripod with a bubble to guarantee a horizontal positioning of the camera. The observations were performed both by the top up to an height of 40 m [Figure 13(a)] and by the bottom of the bridge [Figure 13(b)], in order to individuate the pattern of building and the state of the concrete. Preliminary results are shown in Figure 13, where in the section (a) it is possible to individuate the road joints which are covered by a homogeneous not-damaged asphalt paving, a few centimetres thick. They are characterized by a lower temperature, so that in Figure 13(a) they appear as the darkest lineaments. The measurements performed by the bottom of the bridge have instead allowed to highlight the weathered iron bars constituting the concrete armature.

## 5. Conclusions

In this paper we have presented a brief description of the ISTIMES Project by pointing out its novelty and the key elements. As an example of the first outcomes of the project, we have shown preliminary GPR and Infrared thermographic results on the Musmeci bridge. These results are the starting point for the integrated monitoring system that will be implemented during the ISTIMES project. These two techniques have allowed to detect the surface and inner distribution of embedded rebars, enabling to reconstruct the pattern of the building of the bridge, and the state of the concrete in the surface layer.

Within the framework of the ISTIMES project, the GPR techniques will be used to “zoom in” on possible damage or weak zones that have been already identified by the SAR and optic fiber sensor pathfinder surveys. By integrating these wide-ranging techniques, both large and small areas can be monitored quickly at multiple scales and depths.

As a general comment, the complexity and the diversity of the structures to be investigated and the possible “ambiguity” related to the interpretation of the single geophysical technique observations needs an integration of different geophysical methods [39] and their combination with other a priori information or data such as geomorphological, structural, drilling ones. Therefore, a choice of the techniques to be integrated must be properly performed. In the project, we exploit a novel strategy to evaluate the integration preferences and the advantages of combined interpretation consisting in the application of multi-model approach to the examination of near-surface buried targets will be investigated. Furthermore, the comparison of different electromagnetic and electric methods will be carried out by using indicators to reduce observations to one common level, to reveal weak anomalies against the noise background, and to divide discovered combined anomalies to perspective and non-perspective ones.
